# Use of carglumic acid in valproate‐induced hyperammonemia: 25 pediatric cases

**DOI:** 10.1002/jmd2.12131

**Published:** 2020-06-11

**Authors:** Laura María Palomino Pérez, Álvaro Martín‐Rivada, Elvira Cañedo Villaroya, Juan José García‐Peñas, Margarita Cuervas‐Mons Vendrell, Consuelo Pedrón‐Giner

**Affiliations:** ^1^ Section of Gastroenterology and Nutrition Hospital Infantil Universitario Niño Jesús Madrid Spain; ^2^ Section of Pediatrics Hospital Infantil Universitario Niño Jesús Madrid Spain; ^3^ Section of Neurology Hospital Infantil Universitario Niño Jesús Madrid Spain; ^4^ Section of Pharmacy Hospital Infantil Universitario Niño Jesús Madrid Spain

**Keywords:** carglumic acid, hyperammonemic encephalopathy, valproate‐induced hyperammonemia, valproic acid

## Abstract

Hyperammonemic encephalopathy is a rare but potentially dangerous complication of the antiepileptic drug (AED) sodium valproate (VPA). We report a retrospective study of 25 pediatric patients, (15 females [60%]; age: 7.6 ± 4.9 years), with different underlying disorders, who suffered from hyperammonemia due to VPA and who were treated with carglumic acid (CA). The duration of treatment with VPA was 15 ± 1 month, with a dose of 40 ± 16.6 mg/kg/d. VPA blood levels were 75.5 ± 60 mg/L with seven patients being overdosed (>100 mg/L). Twenty‐three patients received concomitant treatment with other AEDs. The initial dose of CA was 100 mg/kg. Subsequently, CA doses of 25 mg/kg were given to 22 patients every 6 hours (average treatment length 2.17 ± 1.1 days) until ammonemia was normalized. In nine patients, CA was used in combination with other drugs to treat hyperammonemia. In all cases, blood ammonia levels were brought under control and symptoms of hyperammonemia resolved. Two hours after CA administration, the average reduction in ammonium levels was 53 ± 29 and 88.6 ± 47.5 μmol/L at 24 hours, resulting in a statistically significant decrease when compared to pretreatment levels. There were no statistically significant differences between sexes, in the presence or not of cognitive impairment or previous carnitine treatment. There were no statistically significant differences when comparing treatment with CA plus ammonia scavengers vs CA alone. In 17 patients (68%) VPA was discontinued and 62% of the patients who maintained treatment had recurrent episodes of hyperammonemia.

**Synopsis**

Carglumic acid significantly reduced ammonia levels in 25 pediatric patients suggesting that it could be an effective treatment of valproate‐induced hyperammonemia.

## 1 INTRODUCTION

The fatty acid derivative sodium valproate (VPA) has been used as an anticonvulsant in patients with epilepsy for more than 40 years.^1^ The drug is generally considered safe; however, hyperammonemia is a frequent idiosyncratic side effect (16%‐52% of patients receiving VPA treatment may have mild elevations in ammonia)^2^ which is often asymptomatic in children (up to 20% of patients treated with VPA).^3^ Although VPA‐induced hyperammonemic encephalopathy (VHE) is rare (<1/10 000), it is more frequent when VPA is used in combination with other antiepileptic drugs (AEDs)^2^, ^4^, ^5^ and it could be a serious condition, that causes a reduction seizure threshold, cerebral edema, and even death.^2^, ^6^

VPA is extensively metabolized in the liver, mostly by ß‐oxidation in the mitochondria as the main metabolic route (40%), with carnitine being responsible for its entry into the mitochondria, as for all fatty acids.^7^ In healthy individuals, upon activation by N‐acetylglutamate (NAG), carbamyl phosphate synthetase I (CPSI) catalyzes the reaction of ammonia with carbonate and adenosine triphosphate to form carbamyl phosphate, which is subsequently converted to urea.^8^

Several studies have strongly suggested that VPA and its reactive intermediate, valproyl‐CoA (VP‐CoA), inhibit NAG synthase (NAGS), the enzyme that catalyzes NAG formation. The subsequent reduction in NAG content would then inhibit the urea cycle since CPSI would not be sufficiently activated to allow conversion of ammonia into carbamylphosphate and ultimately to urea.^9^, ^10^ Moreover, long‐term high‐dose VPA therapy or acute VPA overdose induces carnitine depletion, which could increase the 𝜔‐oxidation route resulting in higher concentration of 4‐en‐VPA, a toxic metabolite that inhibits CPSI.

If hyperammonemia is detected, VPA should be withdrawn immediately, but since the drug has a half‐life of 9.6 hours,^11^ high blood ammonia levels often persist, exposing the patient to the risk of irreversible damage of the central nervous system.

Carglumic acid (CA) is a structural analogue of *N*‐acetyl‐glutamate that has been successfully used in the treatment of hyperammonemia due to NAGS deficiency.^12^, ^13^ CA has also been effective in other inborn error of metabolism (IEM), such as propionic and methylmalonic aciduria,^14^, ^15^ and fatty acid oxidation disorders. In all of them, the inhibition of NAGS impairs the NAG production and may cause hyperammonemia.

Treatment of VPA‐induced hyperammonemia is not an approved use of CA, even though off‐label use for this indication has already been reported, and different clinical practice guidelines suggest that the use of CA in VPA hypermmonemia is available.^6^, ^16^, ^17^

The objective of this study was to describe the efficacy of CA in the VPA‐induced hyperammonemia in our center.

## 2 METHODS

### 2.1 Patients

An observational and retrospective study was carried out in our tertiary pediatric center (patients <18 years old). All patients with VPA‐induced hyperammonemia treated with CA, from January 2006 to February 2018, were included. Demographic and clinical data of the patients were registered, including age, sex, neurologic disease, treatment duration, dose and levels of VPA, use of concomitant AEDs, blood ammonia levels and associated symptoms. Ammonia and VPA levels before CA administration, at 2 and 24 hours after treatment were studied, as well as the use of other drugs to treat hyperammonemia.

The presence of an IEM that could explain the hyperammonemia was ruled out. Glutamine, glutamate citrulline, and acylcarnitine levels before and during VPA treatment are not measured in our routine clinical practice.

Patients were followed up until resolution of symptoms; and the eventual withdrawal of VPA or recurrences of hyperammonemia were recorded.

### 2.2 Statistical analysis

The Mann‐Whitney *U* test was performed to determine the possible statistical association among the decrease in ammonia levels and qualitative variables, including a comparative subgroup analysis to see if the combination of CA with scavengers allowed for a greater decrease of ammonia levels when compared to CA only.

The Spearman's rho test was also performed to determine the correlation between ammonia levels and its decrease 2 and 24 hours after CA administration.

## 3 RESULTS

Twenty‐five patients were included in the study, fifteen of them were girls (60%). The age varied from 5 months to 15 years, with a mean age of 7.6 ± 4.9 years old. Fourteen patients (56%) suffered from cognitive impairment. The indication of VPA was epilepsy in all the patients, with different underlying conditions (Table 1), except for one patient who received it for a psychiatric disease.

**TABLE 1:** Summary of patients

Case	Gender	Age (y)	Underlying disorder	VPA			Symptoms	Ammonia levels (μmol/L)			Other treatments	Time of treatment with CA	VPA withdrawal	Recurrent episodes	Recurrence		
				Time of treatment	Dose (mg/kg/d)	Levels (mg/L)		Basal	After 2 h	After 24 h					Time between episodes	Ammonia levels (μmol/L)	Treatment
1	F	15	Inv.dup. chromosome 15	2 s	12	65	GCS 9	154	108	70	Carnitine, lactulose	2 d	Yes	No			
2	F	14	DNAmt depletion	7 d	30	71	GCS 13	102	60	73	No	3 d	Yes	No			
3	F	3	Sturge‐Weber syndrome	3 y	62	55	GCS 13	99	97	60	No	3 d	Yes	No			
4	F	6	Cryptogenic epilepsy	3 y	62	51	GCS 13	111	80	53	No	4 d	Yes	No			
5	F	7	Myoclonic epilepsy	2 y	42	76	Somnolence. Cold symptoms	102	73	38	Carnitine (100 mg/kg/d; 1 d)	2 d	Yes	No			
6	M	12	Febrile infection‐related epilepsy syndrome	3 m	43	42.8	Somnolence. Vomiting	150	127	69	No	Single dose	No	Yes	4 m	85	Carnitine (60 mg/kg/d)
7	M	3	Benign familial infantile epilepsy	1 m	22	81.4	Somnolence.	92	88	77	No	2 d	Yes	No			
8	M	6	Autism and epilepsy	1 m	43	45	Somnolence. Vomiting	222	155	77	No	2 d	Yes	No			
9	F	5	Focal cortical dysplasia	7 d	30	97.4	GCS 13	183	59	74	Carnitine	2 d	Yes	No			
10	M	1	Dup. 16p	4 d	40	92	Somnolence. Vomiting	379	74	48	No	2 d	No	Yes	3.5 y	73	None
11	F	11	Focal cortical dysplasia	12 m	30	104	No	110	48	59	Carnitine (70 mg/kg/d; 2 d)	2 d	Yes	No			
12	M	14	47 XYY chromosomopathy	5 d	40	69	Somnolence. Vomiting	172	79	75	Carnitine (60 mg/kg/d; 3 d)	3 d	Yes	No			
13	M	5 m	Sturge‐Weber syndrome	3 d	75	298	Somnolence. Vomiting	219	159	85	Carnitine (100 mg/kg/d;2 d)	2 d	Yes	No			
14	M	13	Cryptogenic epilepsy	1 m	40	110	Somnolence. Vomiting	92	58	70	No	2 d	Yes	No			
15	F	1	Cryptogenic epilepsy	3 d	30	105	Somnolence	104	91	54	No	Single dose	Yes	No			
16	M	4	Focal cortical dysplasia	1 m	40	91	Somnolence. Ataxia	85	63	64	No	6 d	Yes	No			
17	F	2	Del 2q 24.3‐q31.1	2 y	58	90	Somnolence. Diarrhea	151	145	40	No	1 d	No	Yes	5 m	105	None
18	F	3	Sturge‐Weber syndrome	3 d	2	102	Somnolence	183	122	74	Carnitine (150 mg/kg/d; 2 d)	1 d	Yes	No			
19	F	9	Limbic encephalitis	7 d	42	28	Somnolence. Confusion	153	48	26	Arginine. Sodium phenylbutirate	1 d	Yes	No			
20	F	3	Cryptogenic epilepsy	18 m	60	90	Somnolence	99	103	62	No	3 d	No	Yes	1 m	111	CA*
21	F	2	Cryptogenic epilepsy	15 m	60	60	Somnolence	173	113	52	No	2 d	No	No			
22	M	13	Dravet syndrome	12 y	20	110	Somnolence	121	99	91	No	Single dose	No	Yes	1 m	107	None
23	F	14	Psychiatric disorder. VPA and alcohol intoxication	2 y	15	110	Somnolence	224	159	62	Carnitine	2 d	No	No			
24	M	14	Cerebral palsy secondary to meningitis	2 y	20	40	Somnolence	153	105	34	No	2 d	Yes	No			
25	F	10	Agenesis of corpus callosum	6 y	33	75.8	No	149	145	79	No	2 d	No	No			

Abbreviations: GCS, Glasgow Coma Scale; VAP, valproate.

The duration of treatment with VPA ranged from 2 days to 6 years, with a mean length of 15 ± 1 month. The mean dose of VPA was 40 ± 16.6 mg/kg/d, and only five of the patients were receiving a dose over the maximum habitual dose (60 mg/kg/d). The mean VPA blood levels were: 75.5 ± 60 mg/L (therapeutic range 50‐100 mg/L), seven patients (28%) were overdosed (>100 mg/L); only two of them had a dose over the maximum usual dose.

Twenty‐three patients (92%) received concomitant treatment with other AEDs (Table 2), most of them with polytherapy. Ammonia levels prior hyperammonemic episodes were normal in all patients.

**TABLE 2:** Combination with other AEDs in patients' treatment

	VPA	TPM	CLB	PB	CBZ	OXC	STP	LEV	ZNS	RUF	LTG	PHT	VGB	CPZ
Patient 1	•	•	•											
Patient 2	•		•	•										
Patient 3	•	•				•								
Patient 4	•		•				•							
Patient 5	•		•					•						
Patient 6	•			•	•				•					•
Patient 7	•													
Patient 8	•		•											
Patient 9	•					•		•						
Patient 10	•	•						•						
Patient 11	•		•						•	•				
Patient 12	•		•						•					
Patient 13	•							•						
Patient 14	•		•						•		•			
Patient 15	•					•		•						
Patient 16	•		•	•		•		•				•		
Patient 17	•	•	•					•						
Patient 18	•					•						•		
Patient 19	•		•	•				•				•		
Patient 20	•		•					•	•					
Patient 21	•	•	•										•	
Patient 22	•		•				•							
Patient 23	•													
Patient 24	•													•
Patient 25	•							•	•					•

Abbreviations: AEDs, antiepileptic drugs; CBZ, carbamazepine; CLB, clobazam; CZP, clonazepam; LEV, levetiracetam; LTG, lamotrigine; OXC, oxcarbazepine; PB, phenobarbital; PHT, phenytoin; RUF, rufinamide; STP, stiripentol; TPM, topiramate; VPA, valproic acid; VGB, vigabatrin; ZNS, zonisamide.

Clinically, only two patients were asymptomatic, and the hyperammonemia was detected in a blood routine test. One patient had impaired consciousness (Glasgow Coma Scale of nine points), one suffered ataxia, and the others drowsiness, somnolence, and vomiting.

The transaminase values were analyzed at the time of hyperammonemia, with a mean aspartate aminotransferase of 32.3 ± 9.4 U/L (normal values [NVs]: 28‐64), alanine aminotransferase: 18.6 ± 8.6 U/L, (NV: 15‐48) and gamma‐glutamyl transferase: 38.5 ± 43.1 U/L (NV: 8‐30).

The average ammonia level in all patients (n = 25) before treatment with CA was 151.2 ± 63.9 μmol/L (range: 85‐379).

All patients received an initial dose of CA of 100 mg/kg (except for one with a dose of 70 mg/kg), and 22 patients (87%) received maintenance treatment of 25 mg/kg/6 h (time average: 2.17 ± 1.1 days) until ammonemia was normalized. Symptoms experienced by patients due to hyperammonemia resolved within less than 24 hours.

**FIGURE 1:**
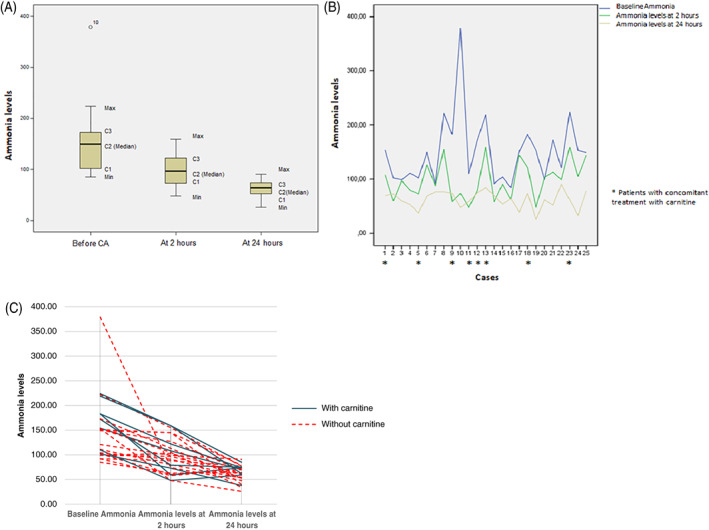
Ammonia levels (μmol/L) before carglumic acid (CA) administration, at 2 and 24 hours after treatment. A, Box plots showing whole cohort of patients with quartiles represented by boxes. Maximum and minimum values are represented by whiskers. B, Reduction of ammonia in the individual patients. Patients with concomitant treatment with carnitine are represented with a star. C, Reduction of ammonia in the individual patients. Patients with concomitant treatment with carnitine vs patients with only CA

Ammonia levels were analyzed both 2 and 24 hours after CA treatment in all patients (n = 25). Two hours after CA administration, the mean ammonia level was 98.8 ± 35.2 μmol/L and the mean reduction was 53 ± 29 μmol/L (35%). Twenty‐four hours after CA administration, the mean ammonia level was 62.6 ± 16.4 μmol/L and the reduction 88.6 ± 47.5 μmol/L (58.6%). Both differences were statistically significant (*P* < .01; Figure 1A).

VPA levels 24 hours after CA administration were only determined in 12 patients (mean: 75.9 ± 61.6 mg/L) and in nine patients 48 hours after (mean 52.9 ± 43 mg/L).

There were no statistically significant differences between sexes in terms of:

1. Decrease in ammonia levels 2 hours after CA administration (*Z*: −0.41, *P*: .68)
2. Presence or not of cognitive impairment (*Z*: −0.78, *P*: .43)
3. Previous carnitine use (*Z*: −1.48, *P*: .14).

There was no statistical correlation in ammonia reduction at 2 hours (*r*: .29, *P*: .16) nor at 24 hours (*r*: −.15, *P*: .95) with the magnitude of the previous levels.

In nine patients, other therapies (ammonia scavengers) in addition to CA were also used to treat hyperammonemia: carnitine (n = 7), carnitine + lactulose (n = 1), phenylbutyrate + arginine (n = 1). When comparing these patients with those who received only CA, there was no difference in the decrease in ammonia levels at 2 or at 24 hours (Figure 1B,C).

In 17 patients (68%), VPA was withdrawn. Five out of eight (62%) who maintained treatment with VPA had recurrent episodes of hyperammonemia, that were treated as shown in Table 1. Patient number 20 required outpatient treatment with CA for recurrent hyperammonemia episodes due to her reluctance to discontinue VPA treatment, which could result in more epileptic seizures.

Recurrent hyperammonemic episodes were not observed in patients who had VPA treatment discontinued.

Determination of serum amino acids and acylcarnitine profile, and urine organic acids levels were performed in 17 patients in order to rule‐out a possible IEM that would justify the current hyperammonemia episode. In the other 8, it had already been performed due to their pathology. They were normal in all of them.

## 4 DISCUSSION

VPA has been associated with hyperammonemic encephalopathy, a risk which is obviously increased in patients with urea cycle disorders, with OTC deficiency being the most common.^6^, ^18^ It is therefore recommended to determine blood ammonia levels and liver function before starting treatment with VPA. VPA is generally considered safe, but can cause side effects like weight gain, dyspepsia, peripheral edema, fatigue, dizziness, drowsiness, hair loss, headaches, nausea, sedation, and tremors. Less frequently, there have been reports of life‐threatening conditions such as hemorrhagic pancreatitis, coagulopathies, bone marrow suppression, coagulation disorders, and hepatotoxicity.^3^, ^19^, ^20^

In addition, hyperammonemia is a frequent idiosyncratic side effect of VPA therapy that is mostly mild and asymptomatic in children; however, although VHE is rare, it is potentially fatal.^2^, ^6^

In pediatric patients, hyperammonemia is defined as blood ammonia levels above 50 μmol/L.^16^ Hyperammonemic encephalopathy presents clinically as impaired consciousness (seizure, somnolence, lethargy, confusion, and even stupor and coma).^21^, ^22^ Other symptoms include vomiting and nausea, low‐grade fever, ataxic gait, blurred vision, and focal neurological deficit. These symptoms may be difficult to differentiate from the underlying disease itself and they can be misdiagnosed as therapeutic failure instead of an adverse event related to the use of VPA.^7^, ^22^, ^23^ Therefore, when patients in treatment with VPA have an acute or progressive encephalopathy, it is recommended to test for high blood ammonia levels.^8^ Liver function tests are usually normal and the electroencephalogram may show epileptic activity that normalizes with progressive clinical improvement of VHE and decreased ammonia levels.^22^

Previously published studies have identified the female sex, age (≤3 years), symptomatic generalized epilepsy, and the concomitant use of phenytoin, phenobarbital, topiramate, zonisamide, levetiracetam, or acetazolamide as significant risk factors for VHE.^2^, ^3^, ^4^, ^5^, ^18^ In particular, concomitant use of phenytoin is most likely to cause hyperammonemia, as it increases the activity of cytochrome P450 enzymes and uridine diphosphate glucuronosyltransferases, which are involved in metabolism of VPA.^24^ Moreover, mental retardation has been suggested as a risk factor for VHE.^20^ In our study, a relation between the decrease of hyperammonemia and sex, age or presence or not of cognitive impairment was not found; however, most of the patients who required CA were using VPA concomitantly with other AEDs (92%) and ammonia levels in patients receiving VPA in polytherapy were higher than those receiving it in monotherapy.^24^

The relationship between blood ammonia levels, daily dosage of VPA, and the risk to develop VHE has been somewhat controversial. Although some studies report a positive correlation between ammonia levels and the dosage and serum levels of VPA,^18^, ^22^, ^24^ the daily dosage of VPA, length of VPA treatment, serum VPA levels and serum ammonia levels do not appear to correlate with onset or severity of VHE. In fact, in most published cases, serum VPA levels are usually within the normal therapeutic range (50‐100 mg/L).^6^, ^8^, ^20^, ^21^, ^22^, ^23^ Similarly, we observed that most of our patients (Table 1), had VPA levels in the therapeutic range and this was not related with the decrease in ammonia levels.

We report 25 patients with VPA‐induced hyperammonemia who were successfully treated with CA. To the best of our knowledge, this represents the largest series of this type of patient treated with CA. All patients, regardless of underlying condition, responded to CA with a significant reduction in ammonia levels and clinical improvement within 2 hours of treatment. These findings cannot be explained solely by discontinuation of VPA, since it has a half‐life of 9.6 hours,^11^ but it is also likely due to an acceleration of ureagenesis due to CA.^25^ The mean time necessary to normalize ammonemia was 2.16 days. In a retrospective, phase 3b study that included patients with hyperammonemia secondary to an organic aciduria, the mean time to normalization of plasma ammonia after CA administration was also 2 to 3 days.^15^, ^26^

The use of carnitine (100‐150 mg/kg/d) to control ammonia levels can be useful, but of limited efficacy in patients without liver failure.^27^ In our study, we did not find statistically significant differences, in terms of greater or faster reduction in ammonia levels, when comparing treatment with CA alone vs CA  + carnitine or other ammonia scavengers. However, our group of patients treated with ammonia scavengers is small and heterogeneous.

Therefore, based on these findings, we believe that in VPA‐induced hyperammonemia, treatment with CA restores the function of the urea cycle, reducing hyperammonemia rapidly and that its use is safe and of first choice. In our country, treatment is available and financed by the National Health System. This treatment should be accompanied by the withdrawal of VPA if possible and the reduction of protein intake.

Hyperammonemia in a child receiving VPA forces to rule out a metabolic pathology when it has not been discarded before. Recurrences were common in patients who maintained VPA treatment (five out of eight patients, 62%). Our results suggest that CA could be an option to maintain normal ammonemia in patients with VPA‐induced hyperammonemia for whom stopping VPA is not feasible, as proposed by other authors.^28^ Moreover, long‐term treatment with CA has been suggested to be effective and well tolerated in patients with organic acidurias.^29^, ^30^

Potential side effects of CA include vomiting, diarrhea, stomach pain, fever, or headache and they were not observed, or at least recorded, in our patients.

In conclusion, CA appears to be safe and effective in the treatment of VPA‐induced hyperammonemia. Based on these findings, we suggest the use of CA as treatment of hyperammonemia due to inhibition of NAGS by VPA.

## CONFLICT OF INTEREST

The authors declare no conflicts of interest.

## AUTHOR CONTRIBUTIONS

Dr C. Pedrón‐Giner had the original idea and contributed to planning the research design, methods, interpretation of the findings, and preparation of manuscript. Dr Á. Martín‐Rivada and Dr Laura María Palomino Pérez contributed to planning the research design, carrying out all aspects of the methods and statistical analysis and writing the majority of the manuscript. Dr E. Cañedo Villaroya contributed to planning the research design, methods, interpretation of the findings, and preparation of manuscript. Dr J. J. Garcia‐Peñas and Dr M. Cuervas‐Mons Vendrell contributed to planning the research design and planning the methods. All authors have been involved in drafting the article and have expressed their agreement to submission.

## References

[1] Löscher W . Basic pharmacology of valproate: a review after 35 years of clinical use for the treatment of epilepsy. CNS Drugs. 2002;16(10):669‐694. 10.2165/00023210-200216100-00003. 12269861

[2] Kasapkara ÇS , Kangin M , Feryal F , et al. Unusual cause of hyperammonemia in two cases with short‐term and long‐term valproate therapy successfully treated by single dose carglumic acid. J Pediatr Neurosci. 2013;8(3):250‐252. 10.4103/1817. 24470826 PMC3888049

[3] Gerstner T , Buesing D , Longin E , et al. Valproic acid induced encephalopathy—19 new cases in Germany from 1994 to 2003—a side effect associated to VPA‐therapy not only in young children. Seizure. 2006;15(6):443‐448. 10.1016/j.seizure.2006.05.007. 16787750

[4] Latour P , Biraben A , Polard E , et al. Drug induced encephalopathy in six epileptic patients: topiramate? valproate? or both? Hum Psychopharmacol. 2004;19(3):193‐203. 10.1002/hup.575. 15079854

[5] Lokrantz CM , Eriksson B , Rosén I , et al. Hyperammonemic encephalopathy induced by a combination of valproate and pivmecillinam. Acta Neurol Scand. 2004;109(4):297‐301. 10.1046/j.1600-0404.2003.00227.x. 15016014

[6] Fernández Colomer B , Rekarte García S , García López JE , Pérez González C , Montes Granda M , Coto Cotallo GD . Valproate‐induced hyperammonemic encephalopathy in a neonate: treatment with carglumic acid. An Pediatr (Barc). 2014;81(4):251‐255. 10.1016/j.anpedi.2013.09.015. 24315420

[7] Vázquez M , Fagiolino P , Maldonado C , et al. Hyperammonemia associated with valproic acid concentrations. Biomed Res Int. 2014;2014:217269‐217267. 10.1155/2014/217269. 24868521 PMC4020540

[8] Segura‐Bruna N , Rodriguez‐Campello A , Puente V , Roquer J . Valproate‐induced hyperammonemic encephalopathy. Acta Neurol Scand. 2006;114:1‐7. 16774619 10.1111/j.1600-0404.2006.00655.x

[9] Coude FX , Grimber G , Parvy P , Rabier D , Petit F . Inhibition of ureagenesis by valproate in rat hepatocytes. Role of N‐acetylglutamate and acetyl‐CoA. Biochem J. 1983;216(1):233‐236. 6418145 10.1042/bj2160233 PMC1152491

[10] Aires CC , van Cruchten A , Iljist L , et al. New insights on the mechanisms of valproate‐induced hyperammonemia: inhibition of hepatic N‐acetylglutamate synthase activity by valproyl‐CoA. J Hepatol. 2011;55:426‐434. 21147182 10.1016/j.jhep.2010.11.031

[11] Redenbaugh JE , Sato S , Penry JK , Dreifuss FE , Kupferberg HJ . Sodium valproate: pharmacokinetics and effectiveness in treating intractable seizures. Neurology. 1980;30(1):1‐6. 10.1212/WNL.30.1.1. 6985719

[12] Hinnie J , Colombo J , Wermuth B . N‐acetylglutamate synthetase deficiency responding to carbamylglutamate. J Inherit Metab Dis. 1997;20(6):839‐840. 9427158 10.1023/a:1005344507536

[13] Morris A , Richmond S , Oddie SJ , et al. N‐acetylglutamate synthetase deficiency: favourable experience with carbamylglutamate. J Inher Metab Dis. 1998;21:867‐868. 9870213 10.1023/a:1005478904186

[14] Gebhardt B , Dittrich S , Parbel S , Vlaho S , Matsika O , Bohles H . N‐carbamylglutamate protects patients with decompensated propionic aciduria from hyperammonaemia. J Inherit Metab Dis. 2005;28(2):241‐244. 10.1007/s10545-005-5260-7. 15877213

[15] Blair H . Carglumic acid in hyperammonaemia due to organic acidurias: a profile of its use in the EU. Drugs Ther Perspect. 2019;35:101‐108.

[16] Grupo de Consenso de Lisboa 2006 y Madrid 2007 . Protocolo Hispano‐Luso de Diagnóstico y Tratamiento de las Hiperamonemias en Pacientes Neonatos y de más de 30 días de vida. 2nd ed. Madrid: Editorial Ergón; 2009:46.

[17] Pintos Morell G , Castiñeiras Ramos D , Puig R , et al. Protocolo de diagnóstico y tratamiento de los trastornos del ciclo de la urea In: Ortega G, ed. Protocolos de Diagnóstico y Tratamiento de los Errores Congénitos del Metabolismo. 2nd ed. Madrid: Ergón; 2018:1‐25.

[18] Tseng Y‐L , Huang C‐R , Lin C‐H , et al. Risk factors of hyperammonemia in patients with epilepsy under valproic acid therapy. Medicine (Baltimore). 2014;93(11):e66 10.1097/MD.0000000000000066. 25192484 PMC4616274

[19] Gerstner T , Bell N , König S . Oral valproic acid for epilepsy—long‐term experience in therapy and side effects. Expert Opin Pharmacother. 2008;9(2):285‐292. 10.1517/14656566.9.2.285. 18201150

[20] Cheng M , Tang X , Wen S , Yue J , Wang H . Valproate (VPA)‐associated hyperammonemic encephalopathy independent of elevated serum VPA levels: 21 cases in China from May 2000 to May 2012. Compr Psychiatry. 2013;54(5):562‐567. 10.1016/j.comppsych.2012.11.001. 23246073

[21] Verrotti A , Trotta D , Guido M , et al. Valproate‐induced hyperammonemic encephalopathy. Metab Brain Dis. 2002;17(4):367‐373. 10.1016/j.revmed.2010.06.010. 12602513

[22] Chopra A , Kolla BP , Mansukhani MP , Netzel P , Frye MA . Valproate‐induced hyperammonemic encephalopathy: an update on risk factors, clinical correlates and management. Gen Hosp Psychiatry. 2012;34(3):290‐298. 10.1016/j.genhosppsych.2011.12.009. 22305367

[23] Twilla JD , Pierce AS . Hyperammonemic encephalopathy due to valproic acid and topiramate interaction. Case Rep Psychiatry. 2014;2014:1‐3. 10.1155/2014/410403. PMC4127233 25136470

[24] Yamamoto Y , Takahashi Y , Imai K , et al. Risk factors for hyperammonemia in pediatric patients with epilepsy. Epilepsia. 2013;54(6):983‐989. 10.1111/epi.12125. 23409971

[25] Ah Mew N , Payan I , Daikhin Y , et al. Effects of a single dose of N‐carbamylglutamate on the rate of ureagenesis. Mol Genet Metab. 2009;98(4):325‐330. 10.1016/j.ymgme.2009.07.010. 19660971 PMC2784258

[26] Valayannopoulos V , Baruteau J , Bueno Delgado M , et al. Carglumic acid enhances rapid ammonia detoxification in classical organic acidurias with a favourable risk‐benefit profile: a retrospective observational study. Orphanet J Rare Dis. 2016;11:32. 27030250 10.1186/s13023-016-0406-2 PMC4815113

[27] Gramage Caro T , Vélez‐Díaz‐Pallarés M , Serna Pérez J , Bermejo Vicedo T . Tratamiento con ácido carglúmico de la hiperamoniemia inducida por ácido valproico en un paciente pediátrico. Farm Hosp. 2012;36(5):437‐438. 10.1016/j.farma.2011.08.005. 22858088

[28] Sattar Y , Wasiq S , Yasin W , et al. Carglumic acid treatment of a patient with recurrent valproic acid‐induced hyperammonemia: a rare case report. Cureus. 2018;10(9):e3292. 30443462 10.7759/cureus.3292 PMC6235635

[29] Burlina A , Cazzorla C , Zanonato E , et al. Clinical experience with N‐carbamylglutamate in a single‐centre cohort of patients with propionic and methylmalonic aciduria. Mol Genet Metab Rep. 2016;13(8):34‐40. 10.1016/j.ymgmr.2016.06.007 PMC4949587 27489777

[30] Tummolo A , Melpignano L , Carella A , et al. Long‐term continuous N‐carbamylglutamate treatment in frequently decompensated propionic acidemia: a case report. J Med Case Reports. 2018;12(1):103. 10.1186/s13256-018-1631-1 PMC5911373 29679984

